# Behavioral Cooperation or Conflict of Human Intestinal Roundworms and Microbiomes: A Bio-Activity Perspective

**DOI:** 10.3390/cells14070556

**Published:** 2025-04-07

**Authors:** Meisam Khazaei, Malihe Parsasefat, Aisa Bahar, Hamed Tahmasebi, Valentyn Oksenych

**Affiliations:** 1 School of Medicine, Shahroud University of Medical Sciences, Shahroud 36147-73943, Iran; 2 Biochemistry Department, Faculty of Medicine, Iran University of Medical Sciences, Tehran 14496-1453, Iran; 3 Faculty of Medicine, University of Bergen, 5020 Bergen, Norway

**Keywords:** parasites, gut microbes, microbiome, bacterial iron regulatory, nematodes

## Abstract

Human infections are greatly impacted by intestinal nematodes. These nematodes, which encompass the large roundworms, have a direct impact on human health and well-being due to their close cohabitation with the host’s microorganisms. When nematodes infect a host, the microbiome composition changes, and this can impact the host’s ability to control the parasites. We aimed to find out if the small intestinal roundworms produce substances that have antimicrobial properties and respond to their microbial environment, and if the immune and regulatory reactions to nematodes are altered in humans lacking gut microbes. There is no doubt that different nematodes living in the intestines can alter the balance of intestinal bacteria. Nonetheless, our knowledge about the parasite’s influence on the gut microbiome remains restricted. The last two decades of study have revealed that the type of iron utilized can influence the activation of unique virulence factors. However, some roundworm proteins like P43, which makes up a large portion of the worm’s excretory-secretory product, have an unknown role. This review explores how the bacterial iron regulatory network contributes to the adaptability of this opportunistic pathogen, allowing it to successfully infect nematodes in different host environments.

## 1. Introduction

From the beginning of our lives, we are inhabited by diverse bacteria in multiple bodily tissues, including the skin, intestine, and lungs. The microbiota consists of bacteria, fungi, protozoa, and viruses, among other microorganisms [1]. This review is primarily focused on analyzing the bacterial aspect of the microbiota, with particular emphasis on commensal bacterial communities, and will refer to them as ‘microbiota’. Host genetics play a role in shaping the composition of the microbiota [1]. The composition and variety of microorganisms in the human body are influenced by a combination of environmental elements like how babies are born, breastfeeding, nutrition, and lifestyle choices, as well as genetic differences. Likewise, it can be modified by viruses, fungi, and parasites [2,3,4].

The gut microbiota in humans is vital for nutrient provision, the regulation of epithelial cell maturation, and the orchestration of innate immune mechanisms. Studies indicate that during helminth infections, there are considerable modifications in the composition and abundance of the microbiota within the gastrointestinal system [1,5]. According to some research, the microbiota of individuals suffering from helminth infections shows significant diversity, especially in cases of polyparasitism, indicating that helminth infections contribute to the distinct microbiota patterns found in populations from low- and middle-income countries [6,7]. Research has shown that bacteria can inhabit parasitic nematodes; however, there is a lack of knowledge regarding which specific microbes may have advantageous or detrimental effects on these organisms. It is reasonable to assert that similar dynamics are present between bacteria and parasitic nematodes, which implies that the composition of the microbiota in the host can influence the health of these nematodes in the digestive system [3].

Numerous studies indicate that infections caused by helminths alter the composition of the intestinal microbiome. Antimicrobial peptides (AMPs) found in the excretory and secretory proteins (ESPs) of helminths possess both lethal and non-lethal properties, which can actively reshape the gut microbiota. There may not be direct experimental proof of this effect on microbiota, but it probably interacts with other processes to account for the helminth-induced alterations observed in humans and animals [3,6,8].

Evidence from *T. muris* studies proposes that the major ESP, p43, may influence the host’s immune reaction throughout the infection process. The resemblance of p43 to a segment of the IL-13 receptor subunit alpha-2 allows it to interact with IL-13, a crucial cytokine involved in the expulsion of worms, thereby enhancing their survival [7,9]. Additionally, p43 can attach to matrix proteoglycans, potentially serving as a reservoir for bound p43, which may aid in the retention of IL-13 within the intestinal milieu. The p43 protein is present in every stage of the *T. muris* life cycle, and similar proteins are observed in other Trichuridae parasites, such as *T. trichiura* and *T. suis*. This suggests that these organisms may employ a shared mechanism to influence the immune response of their hosts, thereby ensuring their survival [9,10,11,12,13].

These findings imply that P43 functions as an essential asset in the arsenal of helminths, facilitating their ability to regulate mineral accessibility in the mammalian gut and the conditions of the gut microbiome. It is essential to explore how this influences initial stages of the worm infection, particularly regarding survival and adherence to the intestinal lining [13]. Presently, the epithelial monolayer cultures derived from organoids do not serve as a sufficiently robust model for studying worm attachment; however, with additional refinement, this methodology has the potential to become an invaluable tool for exploring helminth biology [14].

Abundant and crucial, iron serves as a key metallo-nutrient for nearly all organisms. During infection, pathogenic bacteria must overcome an added challenge in acquiring iron due to its sequestration by innate immune components within the host [15,16,17]. This phenomenon is known as ’nutritional immunity’. Nonetheless, the host must obtain important nutrients, including iron, for multiplication to happen [18]. While bacteria are essential for supplying iron, an overabundance of this nutrient can lead to the Fenton reaction, causing the production of harmful hydroxyl radicals when combined with H_2_O_2_ produced during metabolism. Hence, the capacity of harmful microbes to obtain iron from their host surroundings and utilize it in protein synthesis is a crucial factor in causing illness [19,20].

The relationship between pathogens and metals has been a focus of recent research, which has elucidated the mechanisms by which infectious agents obtain iron from the host and could pave the way for new therapeutic targets. Iron is present in small quantities in living organisms, making it difficult for pathogens to access [21]. Consequently, they are required to search for it within protein complexes and inorganic chelates, extracting it through scavenging methods. The iron uptake system (IUS) has been well documented in bacteria and some protozoans, yet remains relatively unexplored, especially in pathogens that do not directly interact with the host’s circulatory system and therefore cannot acquire iron from blood [18,21,22].

Evidence from human studies indicates that the microbiota of the host plays a significant role in helminth infections, potentially either curtailing or enhancing their occurrence. The microbiota communities of individuals who manage to clear helminth infections autonomously show greater similarity than those of individuals who are still infected. Certain bacterial groups may play a role in enhancing resistance to infections among individuals who can clear the infection on their own. There is a lack of knowledge concerning the iron levels in certain roundworm microenvironments and how these organisms obtain iron. Worms release a variety of proteins and factors that can be studied to determine characteristics like antigenicity [2,18,23,24]. Thus, the restricted presence of iron in the environment frequently acts as a limiting factor for the growth of various organisms, including bacteria and certain nematodes. In this review, we focused on the relationship between microbiota and intestinal worms with two different scientific angles. The initial approach will articulate the prevailing knowledge concerning the mutual effects of nematodes and their microbial ecosystems and then continue with focusing on the IUS.

## 2. Interaction Between Roundworm and Bacteria (Overlooking the Significance of the Iron Uptake System)

We know that the microbiome is a population of microorganisms including fungi, protozoa, bacteria, and, viruses living in different parts of the human body, such as the gastrointestinal tract (GI), which are colonized in humans from birth. The interaction between Helminths and bacterial microbiota has recently attracted the attention of researchers [10,12,25].

Throughout evolutionary development, a significant correlation has emerged between nematodes and bacteria, making nematodes ideal candidates for research on host associations [26]. Antibiotic interventions safeguard against the colonization of *T. muris* in mice, and researchers suggest that bacteria provide necessary elements for the successful hatching of *T. muris* ova. On the other hand, bacteria and their metabolites may bolster the mucosal barrier, safeguarding against parasitic invasion and aiding in the host’s repair mechanisms to alleviate disease. This collectively implies that active interactions occur between parasites, hosts, and microbial entities [26,27].

One of the models that has been used since 1970 to study pathogenic bacteria–host interactions is the *Caenorhabditis elegans* [28]. This free-living nematode is beneficial in many ways, including its small body length (1.5 mm adults) and rapid generation time of about three days. In addition, an adult worm can produce approximately 300 offspring at a time, and this nematode has a transparent body that allows researchers to examine it microscopically [29].

### 2.1. Some Bacteria Can Kill the Roundworm

Various pathogenic Gram-positive bacteria of the genera *Enterococcus*, *Staphylococcus*, and *Streptococcus* and Gram-negative bacteria of the genera *Burkholderia*, *Pseudomonas*, *Salmonella*, *Serratia*, and *Yersinia*, can kill *Caenorhabditis elegans* [1,12,30,31,32,33,34].

### 2.2. Gram-Positive Bacteria

In some people, *Enterococcus faecalis* resides in the normal flora of the digestive system and can result in meningitis, endocarditis, and urinary tract infections. The deletion of a section in *fsrB*, a key player in quorum sensing, can mitigate the bacteria’s lethality without affecting their colonization prowess. It was also found that the insertion mutation in *fsrA* (TX5240) and *fsrC* (TX5242), like *fsrB* (TX5266), can attenuate their ability to kill *C. elegans* [35,36].

*Streptococcus pyogenes* has also been reported as a bacterium that can kill worms. This major human pathogen is implicated in a diverse array of pyogenic infections, which encompass tonsillitis, pharyngitis, scarlet fever, and inflammatory skin conditions. The production of hydrogen peroxide is a characteristic of catalase-negative bacteria in oxygen-rich conditions, leading us to hypothesize that the death of *C. elegans* induced by hydrogen peroxide could represent a widespread strategy employed by streptococci and other catalase-deficient bacteria [37]. When bacterial communities do not possess sufficient capacity to degrade H_2_O_2_, nematodes display a shift in behavior. This occurs because environmental H_2_O_2_ can activate ASJ sensory neurons, prompting an escape from H_2_O_2_ while also obstructing the response of several sensory neurons that normally drive movement toward bacteria. The interaction between hydrogen peroxide and bacteria influences the sensory perception of *C. elegans*, thereby modifying the nematode’s behavior to enhance its likelihood of locating an environment that offers both nourishment and protection against hydrogen peroxide [37,38].

*Staphylococcus aureus* is one of the main causes of hospital infections and a large number of skin and visceral infections such as endocarditis, sepsis, pneumonia, and toxic shock syndrome. It has been seen that the *C. elegans* nematode can feed on the *S. aureus* bacteria, which results in nothing but the death of the nematode. In fact, during this feeding process, bacteria accumulate in the digestive system of the worm and cause the death of the worm [34,39,40].

Several *S. aureus* strains have a one-day LT50, which can be said to be a very fast kill for worms. Various pathogenic factors in *S. aureus* cause pathogenicity in mammals, including the quorum-sensing global virulence regulatory system agr and the global virulence regulator *sarA,* as well as alpha-hemolysin [41] and V8 serine protease—all necessary factors for pathogenicity in worms. There are many genes in *C. elegans*, mutations of which can make the worm susceptible to infection with *staphylococcus*, including esp-2/sek-1 and esp-8/nsy-1. All developmental stages of *C. elegans* nematode, including L1-L4 larvae, were killed by *Staphylococcus* [34]. It is reported that mutations in agr locus and *sarA* are effective in *elegans* susceptibility to *staphylococcus* infection [34]. In addition, extracellular factors including α-hemolysin and a secreted nuclease of *Staphylococcus* are important for killing *C. elegans* [42].

### 2.3. Gram-Negative Bacteria

*Pseudomonas aeruginosa* can kill *C. elegans* in two ways: rapidly by toxin-mediated mechanisms and slowly in an infectious process. In the slow model, after colonization of bacteria, no severe disease symptoms are seen in the first 24 h, and the lethality rate is very small and can be ignored [43]. As the exposure process continues, worms gradually stop pharyngeal pumping, lose their mobility, and then die. By evaluating the LT50 (38 h and 48 h), it was found that the L4 larvae stage is much more sensitive than the adult worm [43].

The expression of *gacA*, *toxA*, and *plcS* genes is necessary for *P. aeruginosa* PA14 pathogenicity, and by creating mutations in these genes, the bacterium is not capable of pathogenicity in the mouse-burn model. Exotoxin A is an enzyme that plays an effective role in the mechanism of slowly killing eukaryotic cells, but by weakening this enzyme (LT50 55–70 h), it was found that other factors also play a role in the process of pathogenesis. Therefore, it can be inferred that the slow killing mechanism is probably multifactorial [44,45].

*Salmonella* is a pathogenic Gram-negative bacterium that is pathogenic in humans, and *Salmonella enterica* serovar Typhi causes very serious systemic typhoid fever. Although this serovar is not pathogenic, *Salmonella enterica* serovar *Typhimurium* is lethal to mice. In addition, this serovar (*Typhimurium*) is a good model for studying systemic infections in mice. It has been determined that *S. enterica* serovar *Typhimurium* can be colonized in the digestive system of *C. elegans* [45,46]. To determine whether *Salmonella* is capable of killing *C. elegans, Salmonella typhimurium* was incubated at 25 °C in the medium containing *S. enterica* to feed the culture. The results indicated that the larval stage 4 (L4) and 1-day-old adults died very quickly (the TD 50 was 5.1 d, compared to 9.9 d for control animals on *Escherichia coli* OP50) [46].

As an opportunistic pathogen, *Serratia marcescens* demonstrates a capacity to infect numerous host species. Infection of the *C. elegans* intestine by this Gram-negative pathogen leads to an LT50 of nearly 4 days and full lethality in 6 days. The initial stages of larvae exhibit a strong resistance to infections, whereas the L4 stage and adult forms show a significant vulnerability to such pathogens. The presence of *S. marcescens* in L4s enhances the capacity of *E. coli* cells to traverse the pharynx without lysis, indicating that the pathogen interferes with the pharyngeal grinding mechanism.

The presence of *Burkholderia cenocepacia* in standard growth media leads to a slow mortality rate in *C. elegans*. The application of green fluorescent protein labeling revealed significant intestinal colonization by the bacteria during this slow-killing experiment, with the worms succumbing to death within a timeframe of 1 to 4 days. *B. cenocepacia* demonstrated total mortality within 24 h when exposed to a high-osmolarity medium, and no colonization was observed during this rapid killing phase. The presence of diffusible products from *B. cenocepacia* in conditioned media resulted in some mortality under conditions of high osmolarity; interestingly, certain worms exhibited immobility and seemed lifeless at the 4 h mark, yet they showed signs of recovery by the 24 h point.

The etiological agent of bubonic plague, *Yersinia pestis*, and the closely related *Y. pseudotuberculosis*, known for less severe infections, produce biofilms that adhere to the head of *C. elegans*. A polysaccharide-rich biofilm layer coats the mouth, preventing feeding and consequently stunting growth. *Y. pestis* employs a strategy that involves colonizing and obstructing the feeding of fleas, its vector, thereby enhancing its transmission to mammals. The synthesis of polysaccharides, governed by the hms genes of *Y. pestis*, is essential for nematode biofilms obstruction. Mutations in the *srf-2*, *srf-3*, and *srf-5* genes of *C. elegans* alter the worm’s surface properties, leading to the inhibition of biofilm adhesion.

### 2.4. Effect of Roundworm on Bacterial Life

Bacteria and intestinal nematodes have had friendly interactions for a long time, and the role of this cooperation in the survival of each cannot be ignored. In a study, it was shown that the nematode *Trichuris* cannot even hatch without the presence of intestinal bacteria, let alone be pathogenic [1,47].

According to the last research, *T. muris* eggs were incubated in an environment containing explants of mouse cecum that contained substantial numbers of bacteria at 37 degrees for 30 min. To prove the role of the presence of bacteria in egg hatching, the eggs were incubated in an environment containing *E. coli*. In the presence of these bacteria, the hatching process was seen at a level similar to the presence of gut explants. Next, filtration was used to remove *E. coli* from the environment. The results indicated the role of the structural component of the bacteria, in the egg hatching process [47,48,49].

The process of *T. muris* egg hatching commences as a result of the interaction with the host’s gut microbiota, specifically at a temperature that optimally reaches 37 °C. This process activates the quiescent L1 larva in the egg, which then carries out broad and localized movements, causing its head to push through a polar plug. As the larvae move, there is a marked increase in egg volume resulting from endosmosis, coupled with a growth in the size of the plug at the relevant pole [50,51]. The pellicula ovi (PO) expands over the polar plug, withstanding pressure until the larval stylet breaches the permeability barrier membrane (PBM) and the electron-dense parietal coating (EdPC) right before the insect hatches. The leading segment of the L1 larva rapidly pushes through the cracked plug, while the rest of the larva gradually makes its way out of the egg through the constricted end [52,53].

**Figure 1:**
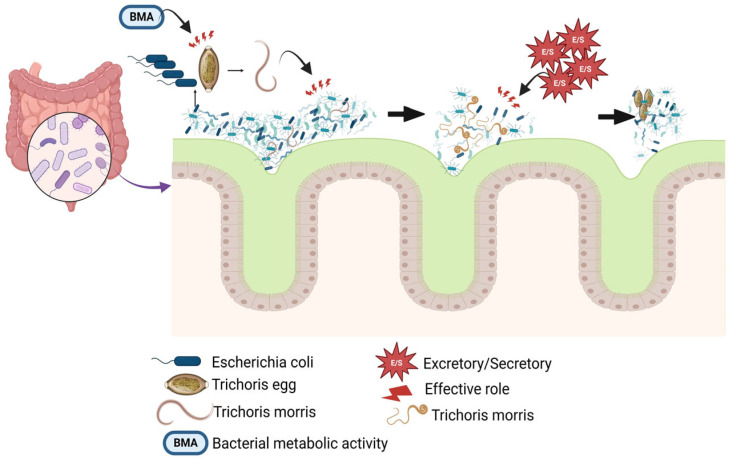
Potential mechanisms of the interaction between *Trichuris* and the host intestinal microbiota. Figure 1 shows that after consuming the infective form of *Trichuris*, certain bacterial metabolic activity, like *Escherichia coli*, can enhance egg hatching, aiding in the infection’s establishment and the parasite’s growth. Eggs and larvae can influence the host microbiota and trigger an inflammatory response. The parasite’s presence in the intestine leads to changes that enhance oxygen levels and reduce important anaerobic bacteria, facilitating the growth of facultative aerobes like Enterobacteriaceae, which may contribute to infection severity. Excretory/secretory products from adult *Trichuris* have the potential to modulate immunity and impact the structure of certain bacterial groups in the intestinal microbiome. This figure was generated using BioRender software version 04.

The capacity to induce *T. muris* egg hatching using a single bacterial species in vitro has facilitated research on the interactions between eggs and bacteria, as well as the molecular processes involved in whipworm egg hatching. Some research revealed that for *T. muris* eggs to hatch, they must come into direct contact with *E. coli*, which congregates around the egg’s polar plug, a process supported by type-1 fimbriae [45,54]. Moreover, Figure 1 reveals that for *T. muris* to hatch, bacterial metabolic activity is necessary, which is triggered by *E. coli* but not by *S. aureus*, and this process is linked to arginine biosynthesis. In conclusion, researchers have depicted the preliminary steps of an asymmetric breakdown of the egg polar plugs following contact with *E. coli* [53,55]. Some researchers recently highlighted that chitinases in the eggs of *T. muris* and *Trichuris trichiura* could be responsible for degrading chitin–protein complexes in the plug, as demonstrated through their experiments with *E. coli* and *S. aureus*. Research has provided some clarity on the role of bacteria in whipworm hatching, yet the mechanisms vary by species, indicating a need for further exploration of the interactions between bacteria and whipworm eggs, along with the larval elements that initiate hatching and the disintegration of polar plugs [45,53,56] (Figure 1).

### 2.5. Antimicrobial Peptides Secreted by Pathogenic Nematodes Kill Bacteria

*Ascaris lumbricoides* and *Ascaris suum* are two ancient pathogenic nematodes from the Ascaridae family, which are pathogenic in humans and pigs, respectively. Although the infection rate of *A. lumbricoides* is estimated to be about 1.5 billion, infection with this helminth is not fatal [12,57]. In a study, it was found that cecropins secreted by *Ascaris* can have bactericidal effects. In this research, which was conducted to investigate the effects of scropins secreted by *Ascaris*, scropins P1-P4, which were chemically synthesized and evaluated, and the microbicidal activity was estimated as MBC in 10 mM Tris/HCl buffer. *A. suum* is one of the pathogenic nematodes infecting pigs. Excretory/secretory products (ESPs) and body fluids (BFs) from *A. suum* have antimicrobial activities [57,58,59].

**Figure 2:**
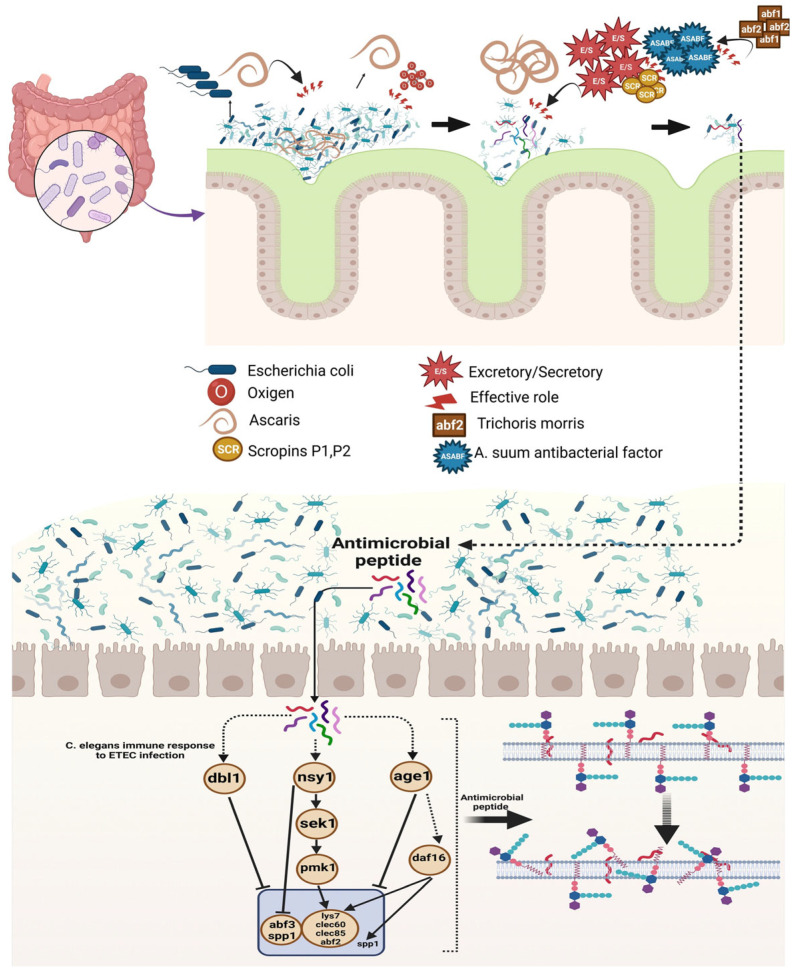
Initial engagements between cationic antimicrobial peptides and bacterial cells occur at the surface level. Cationic antimicrobial peptides are drawn to the anionic components present in the membranes of both Gram-negative and Gram-positive bacteria through a combination of electrostatic and hydrophobic forces. Figure 2 highlights that both daf-2, a gene similar to the human insulin/IGF-1 receptor, and age-1, which is comparable to the human phosphatidylinositol-3-OH kinase (PI3K), show features indicative of a longer lifespan. In the pathways illustrated in Figure 2, the binding of an insulin-like molecule triggers the activation of DAF-2, which then phosphorylates itself and engages its downstream effector AGE-1 to facilitate the conversion of PIP2 into PIP3, while DAF-18 works against this process. The activation of 3-phosphoinositide-dependent protein kinase 1 occurs as a result of PIP3. As a result, the key downstream target DAF-16 undergoes phosphorylation, leading to its inactivation, which prevents its translocation into the nucleus and disrupts the transcriptional regulation of numerous effectors associated with stress resistance, longevity, and fat metabolism. The daf-16 mutation significantly mitigates the impacts caused by the daf-2 and age-1 mutations. The infection of *C. elegans* by bacteria triggers the activation of the p38 MAPK pathway (PMK-1), which subsequently promotes the release of antimicrobial peptides and initiates an innate immune response. The antimicrobial defense in *C. elegans* is regulated by conserved MAPK signaling pathways. Bacterial infections in the intestine activate the p38/PMK-1 signaling pathway through the kinases NSY-1 and SEK-1. Dashed lines illustrate the cascades mentioned in earlier research, whereas solid lines reflect the findings from the current investigation. 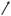 Upregulation; 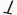, downregulation. This figure was generated using BioRender software version 04.

In some investigations, this property was evaluated against Gram-positive and Gram-negative bacterial strains using the radial diffusion assay. Then, using the crystal violet static biofilm and microcolony assays, the effects on biofilm formation were assessed [60]. We showed in Figure 2 that *A. lumbricoides* (one of the pathogenic species in humans) and *A. suum* can produce cecropin P1 and cysteine-rich peptides, as well as *A. suum* antibacterial factor (ASABF), which has bactericidal activity against either Gram-negative and Gram-positive bacteria, respectively. It can be concluded that this system performs the function of feeding and also defense against infection for nematodes. Antibacterial, bactericidal, and agglutination are three humoral defense activities in the body fluids of *A. suum*. However, Gram-positive bacteria (*S. aureus* and *Bacillus subtilis*) were more sensitive than Gram-negative (*E. coli*) bacteria against these activities [60,61,62].

Three types of humoral defense activity have been identified in *Ascaris* body fluids (antibacterial, bacteriolytic, and agglutinating). Gram-positive bacteria are much more sensitive to antibacterial effects than Gram-negative bacteria. Some yeasts are also sensitive to ASABF [62,63]. Recently, antimicrobial activities in the body fluids of *A. suum* have been reported under the title of ASABF, which is a CSab-type antimicrobial peptide that contains four intramolecular disulfide bridges. In a study, the antibacterial activity of purified ASABF was evaluated and it was found that *S. aureus*, *Micrococcus luteus*, and *B. subtilis*, which are all Gram-positive, were much more sensitive than Gram-negative bacteria *E. coli* and *Proteus vulgaris* [64,65].

A study was carried out to assess the antibacterial activity of *A. suum* BF and ESP on different bacteria. ESP in adult worms had antimicrobial activity against all strains and no difference was seen between ESP in males and females. While BF obtained from the L4 stage showed more antimicrobial activity than other stages [64,66]. ASABF is widely seen in medically important pathogenic species such as nematodes and hookworms, as well as in genetically important models such as *C. elegans*. It was found that two genes *abf-1* and *abf-2* encode ASABF in *C. elegans*. In all the tests, Gram-positive and negative bacteria and yeasts were sensitive to abf-2 (Gram-positive bacteria were more sensitive), and some bacteria were also resistant [65,67] (Figure 2).

### 2.6. A Brief Attention to the Interaction Between Probiotics and Roundworms

Probiotics are commonly defined as “live microorganisms that, when administered in adequate amounts, confer a health benefit to the host”. The majority of species known to have probiotic properties belong to the genera *Lactobacillus* and *Bifidobacterium*, commonly found in the gastrointestinal tract of humans and animals and thus generally regarded as safe [68,69]. Amongst various possible mechanisms of action, probiotics are believed to exert their effects by production of antimicrobial substances, competition with pathogens for adhesion sites and nutrients, enhancement of host mucosal barrier integrity, and immune modulation. Thus, the beneficial activities of probiotics are attributable to three main core benefits: supporting a healthy gut microbiota, a healthy digestive tract, and a healthy immune system [68].

In *C. elegans* animal models, several studies have been carried out to understand the mechanisms through which probiotics can influence ageing, by activating different longevity signaling pathways related to oxidative stress resistance. Prolongevity and oxidative stress responses in *C. elegans*-fed probiotics are induced via mechanism(s) that can be DAF-2/DAF-16-dependent or a result of a cross-talk among different pathways [68].

## 3. Interaction Between Roundworm and Bacteria (Direct Attention Towards the Iron Uptake System)

Nutritional factors (like iron) may play a significant role in the interaction between helminths and bacteria. Unabsorbed dietary iron travels to the colon, and there is a growing body of evidence suggesting that this may aid in the proliferation of intestinal pathogens. Iron is essential for the growth and development of the vast majority of bacteria in the gut, prompting them to employ diverse strategies to obtain this crucial nutrient, which is commonly found in a state of low or zero solubility as Fe^3+^ [19,70].

Mutations in *E. coli* that impair the fertility of *C. elegans* due to deficiencies in fatty acid synthesis and ethanolamine metabolism will similarly disrupt the normal development of *T. muris* parasites in gnotobiotic mice that harbor these *E. coli* variants. The infection caused by *T. muris* elevated the expression levels of bacterial genes that facilitate the use of ethanolamine, likely as a strategy to enhance its own survival and adaptability. Bacteria play a crucial role in regulating iron, an essential nutrient, which influences the developmental signals received by *C. elegans* and may impact the interactions between helminths and the host’s microbiota. The complex interplay between microbes and their host obscures a clear understanding of the strict regulatory mechanisms that oversee these processes [71,72].

### 3.1. Effect of Iron Uptake System in Bacteria and Microbiome

Recent research has shown a growing connection between the gut microbiome and iron levels, although most studies on the impact of iron on the human gut microbiome have concentrated on infants. Siderophore receptors within a bacterial strain could detect siderophores that are not produced by that particular strain, and the common periplasmic transport systems may prevent the strain from using energy on distinct ABC transporters for each receptor. The ability of bacteria to harbor receptors for xenobiotic siderophores offers significant advantages, allowing them to acquire iron without the need for siderophore production, and potentially expanding their habitat range [19,73].

**Figure 3:**
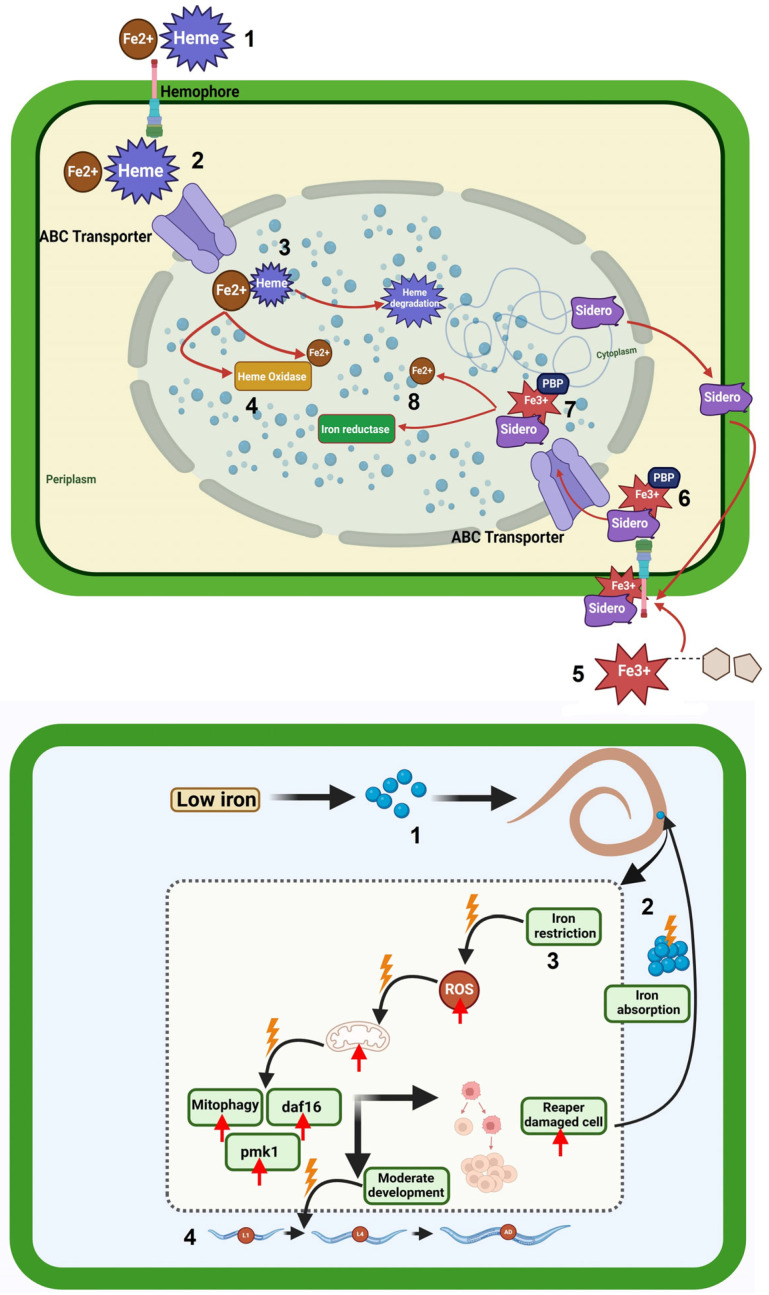
The influence of the iron uptake system on both nematodes and the associated microbiome is a topic of considerable interest and importance in the field of biological research. This system plays a crucial role in the interactions between these microscopic organisms and their surrounding microbial communities, ultimately affecting their growth and development. Bottom figure: The pathways for Hem and ferrous iron absorption operate independently but are closely regulated together. Elevated intracellular iron causes the Holo Fur complex to bind to iron uptake gene regulators, effectively silencing their expression (1). If the iron concentration inside cells is low, Holo Fur liberates Fe^2+^ iron, resulting in the formation of Apo Fur (2). When Apo Fur can’t bind to regulatory sites, it leaves them available, which in turn supports the expression of genes that are crucial for iron uptake (3, 4). To capture ferric iron, bacteria create and secrete siderophores, which are specialized compounds that chelate iron, into the extracellular space (5). The receptors located in the outer membrane, which depend on TonB, recognize the Fe^3+^-siderophore complex, resulting in a shift in the configuration of the channel’s plug domain to enable its internalization (6). The TonB system is energized by ExbB and ExbD through an electrochemical charge gradient along the cytoplasmic membrane, allowing the Fe^3+^ -siderophore complex to be discharged into the periplasm (7). Additionally, binding proteins located in the periplasm facilitate the transfer of the complex to the corresponding ABC transporter, which then moves it into the cytoplasm (8). Below figure: The nematode *C. elegans* faces threats from various pathogens, especially *P. aeruginosa*, while maintaining essential immune responses such as the p38 MAPK pathway (1). *C. elegans’* adaptation to food and pathogens can result in compromises (2), such as diminished reproductive output, accelerated growth, immune system engagement, and depletion of body lipids. Various pathogens attack specific cellular mechanisms and organelles in the host, especially those rich in iron like mitochondria. Acting as the energy factories of cells, mitochondria are key contributors to the generation of reactive oxygen species (ROS) (3). ROS play a role in signaling at physiological levels, but when present in excess, they can damage biomolecules and disturb iron balance (4). This figure was generated using BioRender software version 04.

When oxygen is present and pH is neutral, iron that is not biologically bound is mostly in the insoluble ferric form (Fe^3+^), leading to the formation of unusable oxides or hydroxides. In contrast, under anaerobic or acidic conditions, iron is generally in the soluble ferrous state (Fe^2+^). Bacteria may use direct uptake systems for either form based on the iron’s availability [74,75]. However, free iron, although necessary for organisms, can be toxic due to Fenton reactions, where it reacts with oxidative substances like peroxide, resulting in the formation of harmful radicals such as hydroxyl and superoxide. The dual nature of iron necessitates careful monitoring of its intracellular levels and strict regulation of its uptake. Based on Figure 3, many bacteria employ the ferric uptake regulator (Fur) to sense iron. When it binds to ferrous iron inside the cell, it undergoes a structural shift that allows it to connect with specific genomic sites, ultimately inhibiting the expression of downstream genes. The Fur- Fe2+ complex was once believed to mainly regulate genes for iron uptake, but current knowledge shows it also influences various genes outside of iron metabolism [75,76].

FeoABC is the only ferrous iron uptake system that many bacteria use, but there is still limited knowledge about its functionality. Usually, the Feo system consists of three proteins: FeoA, FeoB, and FeoC (YhgG). The theory suggests that in Gram-negative bacteria, ferrous iron diffuses into the periplasm via unidentified porins and is transported across the cytoplasmic membrane by FeoB, with energy derived from GTP hydrolysis [15]. However, siderophores are employed to obtain ferric iron when iron is scarce. Bacterial operons frequently group genes for siderophore synthesis alongside those for their uptake. Specific receptors on the cell membrane bind siderophores, allowing for their movement into the periplasm in Gram-negative bacteria or the cytoplasm in Gram-positive bacteria. There are several types of bacterial siderophores with different structures, and they can vary in iron affinity, secretion rates, and uptake rates. Enterobactin, a catechol siderophore, binds strongly to ferric iron in lab conditions, but its efficiency in living organisms is lower compared to the α-hydroxycarboxylate siderophore aerobactin [75,77].

Since iron overload results in the generation of reactive oxygen species (ROS) through the activation of the Fenton reaction, iron homeostasis should be tightly regulated. Moreover, the iron imbalance, driven by iron deficiency or iron overload, is connected with many human diseases like metabolic, hematological, and neurodegenerative diseases. Additionally, excess iron introduced by exposure to high concentrations of ferric ammonium citrate (FAC) in *C. elegans* resulted in protein oxidation, upregulated ROS generation, and shortened lifespan. Thus, it is crucial to maintain iron homeostasis to avoid the negative consequences of the disruption of iron metabolism and its distribution [22,78].

### 3.2. Effects of the Iron Uptake System on Nematodes and the Microbiome

For nematodes as well as bacteria, iron is a necessary nutrient. For numerous enzymes involved in vital metabolic processes, it acts as a cofactor. Sufficient availability of iron stimulates the growth and reproduction of bacteria, which may have an indirect effect on nematode populations that depend on bacteria for feeding. Different tactics are used by bacteria to obtain iron from their surroundings. The production of siderophores, which are tiny molecules that chelate iron and make it easier for it to enter bacterial cells, is one typical process [74,79]. Additionally, siderophores can bind to iron in the surrounding environment, reducing its availability to nematodes and other organisms. In the intestine, nematodes—including certain parasitic species—actively consume bacteria. They have chemosensory receptors, which let them recognize siderophores and other chemical cues from bacteria. Nematodes’ intake and dispersion can be affected by the presence of bacterial siderophores, which draw them to regions with higher bacterial concentrations [21,24].

Intense competition between bacteria and nematodes can result from a limited iron supply in the intestine. Some bacterial strains that produce little or no siderophores may be outcompeted by bacteria that use siderophores to scavenge iron. The composition and quantity of bacterial species in the gut environment can be impacted by this competition, which may have an indirect effect on nematode populations [74]. Some pathogenic bacteria can express their virulence genes in response to iron deficiency. This phenomenon dubbed the “iron-withholding defense”, is an immunological reaction of the host intended to limit the growth of germs by securing iron. In reaction, certain bacteria increase the synthesis of virulence factors to overcome the iron restriction and cause illnesses. By feeding on harmful bacteria only, nematodes can help regulate the amount of these bacteria in the intestine by lowering their population [80,81] (Figure 3).

### 3.3. Iron Uptake System and Nematodes Compete with Bacteria

Knowing the complex interactions between nematodes, bacteria, and iron in the gut is essential to understanding the dynamics of microbial populations and how they affect host health. Nematodes have practical techniques for absorbing host iron while competing with microorganisms. Nematodes compete with bacteria for the absorption of host iron through several useful methods. Some of these mechanisms are outlined below.

#### 3.3.1. Secretion of Iron-Chelating Compounds

From bacteria to higher eukaryotes, a common element that restricts an organism’s growth is a lack of iron in the environment. *Pseudomonads* and other bacteria use the tactic of secreting high-affinity iron-binding siderophores (like pyoverdine) to adjust to the low availability of iron. In environments where there is a shortage of iron, these siderophores effectively scavenge iron from the surrounding environment, improving microbial viability [74]. An interesting ecological question is raised by the widespread release of siderophores by bacteria. Because pathogenic bacteria are chemorepellent to predators that feed on bacteria, several microbial siderophores operate as virulence factors against their hosts. For instance, it has been noted that pyoverdine kills *C. elegans* [82,83]. Pyoverdine is released by *P. aeruginosa*, and it was revealed in an interesting finding that *C. elegans* uses the chemosensory receptor Odr-10 to detect and react to its presence. These compounds did not have the expected chemorepellent effect; rather, they served as cues that attracted nematodes. This finding emphasizes the wider influence of these interactions on ecological processes and has important implications for population dynamics and predator behavior [84,85].

#### 3.3.2. Physical Disruption of Bacterial Biofilms

Biofilms, or communities of bacterial cells that colonize natural surfaces and host species, are a phenomenon that can be produced by bacteria. To colonize both biotic and abiotic surfaces, for instance, the opportunistic bacterium *Pseudomonas aeruginosa* secretes different types and quantities of adhesion proteins (like CdrA), extracellular DNA (eDNA), and exopolysaccharides (including Pel, Psl, and alginate) [86].

When *P. aeruginosa* and *Salmonella* biofilms are internalized into the intestine, they can produce certain virulence factors, such as pyoverdine, that can cause *C. elegans* to die. While these effects mostly affect the eating behavior and intestinal infection of *C. elegans*; one important feature of the organism that is yet unknown is whether biofilms can change its motility [87].

Ferric chloride has been found to directly decrease *P. aeruginosa* biofilm growth in various studies. High FeCl_3_ concentrations were shown to lower the biofilm quantity in *P. aeruginosa* cultures grown on polystyrene microtiter plates. Various iron salts, including ferric chloride, were tested by scientists to observe their influence on biofilm formation in *P. aeruginosa*. Moreover, the study included testing the effects of iron salts on biofilms that had already developed. In line with prior research, elevated concentrations of iron compounds, like ferric chloride, result in a reduction in the formation of biofilms by *P. aeruginosa* [19,21,88].

The movement patterns of *C. elegans* and the resulting behavior—known as the “quagmire” phenotype—can be altered by the biofilm matrix. A collection of *P. aeruginosa* mutants that are known to be involved in the synthesis and control of the biofilm matrix were used to study this phenomenon. Psl is a crucial exopolysaccharide that is present in the *P. aeruginosa* biofilm matrix. It has been reported that Psl inhibits nematode motility, which causes *C. elegans* to move less quickly and exhibit less roaming behavior. One important effect of nematode entrapment in the *P. aeruginosa* biofilm matrix was to hinder *C. elegans’* capacity to navigate toward susceptible OP50 biofilms or escape from a repulsive blue-light stimulus [89].

#### 3.3.3. Predation on Bacteria

Some nematodes, such as *T. trichiura* and *A. lumbricoides*, are predacious and feed on bacteria. By consuming bacteria, predatory nematodes use digestive enzymes to break down the bacterial cells and obtain nutrients, such as iron nematodes, and not only reduce the bacterial population but also gain access to the iron present within the bacterial cells. This predation strategy provides nematodes with a direct source of iron for their nutrition [45,90].

#### 3.3.4. Induction of Host Immune Response

Nematodes can manipulate the host immune system to their advantage. They can secrete molecules that modulate the host’s immune response, suppressing the activity of immune cells that would otherwise target and eliminate nematodes. This allows nematodes to establish a prolonged presence within the host, accessing and utilizing iron resources without being efficiently eliminated by the immune system. *Trichinella spiralis*, *Strongyloides stercoralis*, *Brugia malayi,* and *Wuchereria bancrofti* are some nematodes that can use this mechanism [45].

Nematode infections can cause an inflammatory response in the host, which can result in the release of inflammatory mediators and the recruitment of immune cells. Low levels of iron, however, may have an impact on both the duration and intensity of this inflammatory reaction. Studies suggest that low iron levels can attenuate the inflammatory response by reducing the synthesis of pro-inflammatory cytokines such as TNF-α and interleukin-6 (IL-6) [81,91].

In addition, the host immune response to nematode infections frequently divides into Th1 and Th2 responses, which indicate different patterns of immune activation. This Th1/Th2 balance may be impacted by iron shortage, possibly pushing it in the direction of a Th2-type response. Since nematodes have evolved to thrive in surroundings with Th2-skewed immune responses, this change may be beneficial to them. A low iron level can also impair immunological effector pathways that are essential for fighting worm infections [92,93]. Iron-dependent enzymes that produce reactive oxygen species (ROS), which are essential for eliminating pathogens, are one such method. The immune cells’ ability to destroy nematodes can be compromised by low iron availability, which can inhibit their ability to produce ROS. Another note about how nematodes affect the immune system when there is an iron low is that the development of antibodies, which are essential for the immunological response against nematode infections, can be impacted by a lack of iron. The production of immunoglobulins, which include antibodies like IgG and IgA, requires iron. A decrease in antibody production may result from decreased iron availability, which could hinder the host’s capacity to generate a strong humoral immune response against worms [93,94].

**Figure 4:**
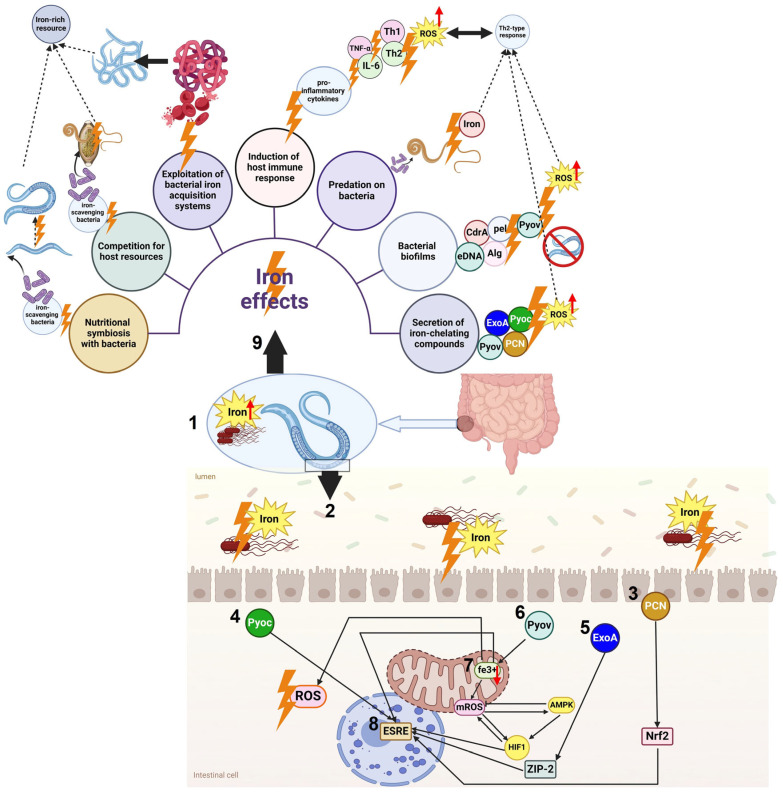
The intricate iron uptake system, often referred to as iron uptake system engages in a competitive struggle against nematodes, which are small, elongated worms, as they vie for resources with bacteria present in the environment. In nematodes, DAF-16 modulates transcription, boosting immunity against stress caused by pathogens and their byproducts. Mammalian Nrf2 is essential for regulating innate immune responses in alveolar and bronchial epithelial cells. Despite the presence of PMK-1 phosphorylation sites on DAF-16, there is minimal overlap in the immune-related gene expressions regulated by PMK-1 and DAF-16, as well as PMK-1, suggesting distinct target effectors (1, 9). The p38 MAPK pathway plays a key role in the increased lifespan seen in daf-2 mutants. When nematodes begin their defense mechanisms against pathogens, *P. aeruginosa* has already established itself in the intestines, disrupting vital cellular processes such as translation and mitochondrial function (2, 3). The key sign of infection is ExoA toxicity, which displays common traits in mammals and nematodes (4). The inhibition of translation by ExoA promotes ZIP-2 expression via translational reduction. The unique and specific characteristics of the ZIP-2 transcriptional pathway have effectively enabled the coevolution of a highly accurate neuronal upregulation, which is crucial for the rapid behavioral avoidance response, in addition to a ZIP-2-dependent antimicrobial reaction that is initiated by the ASJ chemosensory system’s recognition of 1-undecene produced by the PA14 strain (5, 6, 7). In reaction to the harmful impact caused by the pyoverdines secreted by *P. aeruginosa*, there is a notable upregulation of the ESRE network, indicating its heightened activity in the presence of such damage. The increase relies on the transcriptional control of ZIP-2 and various bZIP factors. Monitoring mitochondrial equilibrium is the main function of the ESRE network, which does not engage in direct mediation of antimicrobial effects (8). This figure was generated using BioRender software version 04.

Figure 4 demonstrated that the management of mitochondrial reactive oxygen species (mtROS), which are detrimental byproducts generated by the electron transport chain, plays a vital role in sustaining metabolic equilibrium, maintaining redox balance and promoting longevity. The concept of mitohormesis involves the continuous evaluation and potential initiation of evolutionary pathways, which include HIF1α-mediated transcription and non-transcriptional signaling routes like superoxide dismutase (SOD), mitophagy, and tissue-specific NADPH oxidase (NOX) [95]. To mitigate the levels of harmful ROS, it is important to engage Nrf2-regulated pathways that govern antioxidant and oxidative stress responses, utilizing enzymes associated with peroxiredoxin, thioredoxin, and glutathione. When pathogens invade and cause mitochondrial dysfunction in mammals and nematodes, HIF-1 regulates mtROS levels by increasing the availability of free iron. The importance of this answer is heightened since the *P. aeruginosa* toxin, pyoverdine, leads to a decrease in iron. At the same time, a negative feedback mechanism regulates the production of mtROS via direct control of HIF-1 by AMPK, reducing the harmful impacts of free radicals generated by mitochondria [93,94].

#### 3.3.5. Nutritional Symbiosis with Bacteria

While nematodes compete with bacteria for iron, they can also form symbiotic relationships with certain bacteria that facilitate iron acquisition. Some nematodes establish mutualistic associations with iron-scavenging bacteria, allowing them to access iron sources that would otherwise be inaccessible. These bacteria can produce siderophores or other iron-binding compounds, enhancing the iron availability for both the nematode and the bacteria [95].

#### 3.3.6. Exploitation of Bacterial Iron Acquisition Systems (BIAS)

Nematodes have evolved the capacity to take advantage of bacterial iron acquisition systems. As an example, certain nematodes may imitate the chemical cues that bacteria use to obtain iron from their host. Nematodes can effectively take over the host’s iron transport pathways by generating identical molecules, which puts them in competition with bacteria for the uptake of iron [96].

Nematodes can now access the host’s iron reserves and obtain this vital ingredient for their growth and survival. Nematodes can outcompete bacteria in the hunt for iron because they have essentially evolved mechanisms to alter the host’s iron acquisition pathways. For example, *Necator americanus* and *Ancylostoma duodenale* are two species of hookworms that parasitize humans. These nematodes have evolved a tactic to take advantage of hemoglobin, an iron-rich resource. They generate hemoglobinases, which are enzymes that allow them to break down hemoglobin—the protein in red blood cells that carries oxygen. The nematodes obtain access to the iron reserves in the host’s body by decomposing hemoglobin, which they can then use for self-survival and procreation [97,98].

Macro-autophagy plays a crucial role in the selective degradation of dysfunctional mitochondria, supporting energy and nutritional stability in cells. *P. aeruginosa*’s pyoverdine disrupts mitochondrial iron homeostasis, triggering the ethanol and stress response element (ESRE) network along with a wider mitophagic response to recycle damaged organelles. Notably, mitophagy demonstrates a specific response to the liquid intoxication induced by PA14 and mirrors this response during abiotic iron extraction [84,99]. Furthermore, mitochondria-targeting pyoverdines accumulate in a manner akin to the intestinal LROs in *C. elegans*, possibly as a strategy for sequestration. This reveals that the clearance process chiefly concentrates on shifts in mitochondrial equilibrium, in addition to bolstering the body’s defense against microbes. Evidence supporting this concept includes the fact that the resistance against pathogens that utilize siderophores is improved through the established p38 MAPK pathway, but it depends on the ZIP-2 mechanism within the immune system [1,84,100,101].

#### 3.3.7. Competition for Host Resources

Nematodes confront competition from bacteria not only for iron but also for other vital substances found in the host that are necessary for their survival and ability to reproduce. Nematodes have an indirect impact on bacterial populations’ availability of iron by outcompeting bacteria for these resources. This edge over competitors makes it easier for the nematodes to obtain and use host iron [101,102].

For *C. elegans*, effective mitochondrial activity in epithelial cells is important for energy and metabolic regulation. The assessment of mitochondrial activity relies on the collaboration of pathways that sense unfolded proteins, ROS, low oxygen, and ATP deficiency. Thus, the capacity to either hinder or safeguard mitochondrial activity is vital in the conflict between *P. aeruginosa* and *C. elegans*. The interplay includes detailed mechanisms associated with the pathogen’s secreted factors and the host’s protective responses [1,103].

As shown in Figure 4, a critical pathway encompasses a group of genes that are stress-responsive and include the ethanol and ESRE motif. A high level of conservation characterizes this network, which recognizes mitochondrial impairment resulting from a diverse array of abiotic challenges. The ESRE network is activated in response to the harm inflicted by *P. aeruginosa* pyoverdines. The ESRE network is believed to primarily oversee mitochondrial stability rather than directly affecting antimicrobial responses [92,101].

The latest findings on the roundworm–microbiota interaction demonstrate how infectious agents exploit the host’s iron resources, pointing towards new avenues for therapeutic development. Living organisms contain minimal amounts of iron, making it difficult for pathogens to access and necessitating their acquisition from protein complexes and inorganic chelates. Two types of iron sources, transferrin-bound and non-transferrin-bound, are crucial for pathogens [92,104,105]. In the blood, transferrin is a glycoprotein that can bind a maximum of two Fe^3+^ ions. Within non-transferrin-bound iron, there are feeble complexes, such as albumin, and more resilient complexes. One of the most familiar proteins is hemoglobin, the pigment in red blood cells that facilitates the transfer of oxygen to tissues from the lungs. Mainly situated in muscle tissue, myoglobin binds iron more effectively than hemoglobin, a protein with similar characteristics. Haptoglobin binds to free hemoglobin with oxidative reactivity to prevent its oxidative effects. Haemopexin functions as a carrier of haem and the associated metal ions, transporting them to the liver [92,106].

Infection triggers an ongoing contest for iron between the host and the invading pathogen, prompting the host to develop tactics to deplete the environment of this essential element and hinder its access by the pathogen. Due to this, harmful bacteria interpret the lack of iron as an indication that they have entered a vertebrate host. To reduce the amount of accessible iron, the host utilizes strategies such as the secretion of transferrin and lactoferrin, which can sequester Fe^3+^. Due to the scarcity of iron, pathogens have evolved to possess a greater virulence by either binding to or degrading iron carriers [22,106].

Research on *Entamoeba histolytica* found that a virulent strain needed lower iron levels to survive than an attenuated strain due to improved iron binding and absorption abilities. The protein lipocalin, released by intestinal epithelial cells, can bind to siderophores to hinder bacteria’s iron absorption. Pathogenic *Salmonella* has altered its siderophore structure to avoid being bound by lipocalins, thus evading this immune defense mechanism. Lacking lipocalin 2 (LCN 2) in wild-type mice prevented the systemic spread of bacteria by inhibiting siderophores, unlike transgenic mice without LCN 2 who succumbed to the infection [107] (Figure 4).

### 3.4. The P43 Protein and Killing Bacteria Role in High Levels of Iron

Iron uptake is a topic that has been thoroughly researched in bacteria and certain roundworms, yet there is a notable gap in understanding this process, particularly in pathogens that do not have access to the host’s blood for iron procurement. The iron levels in the microenvironment of *T. muris* and how the worm absorbs iron are poorly understood [108]. Worms release a collection of proteins and substances that can be studied for characteristics like antigenicity. The excretory/secretory products of *T. muris* are not well understood, but studies have demonstrated their ability to induce protective immunity when given as whole worm extracts or purified excretory/secretory products. Culturing live worms for several hours in a culture medium is a method to obtain *T. muris* E/S. P43, a 43 kDa protein, is the predominant element found in the E/S of *T. muris* and various Trichuris species, with identified functions including pore formation in lipid bilayers and similarity to IFN-γ [105,109].

Within the human body, *T. trichiura*, a member of the widely distributed *Trichuris* genus, occupies a particular biological niche. The proximal colon of the large intestine and the cecum’s epithelial layer are where these parasites settle down [26]. *Trichuris* infections are known for their protracted nature, as these parasites can live for a very long time as chronic infections. Interleukin-13 (IL-13) frequently mediates the protective immune response against gastrointestinal-dwelling nematode infections. The most prevalent protein in the whipworm *T. muris*’s mature secretions is a protein known as p43. Glycosaminoglycans (GAC) and the host cytokine IL-13 may interact with p43. Studies on binding have verified that p43 binds to heparan sulfate (HS) and IL-13 with significant affinity [26,108,109].

Furthermore, there is evidence to suggest that p43 has immunomodulatory features. Studies performed inside organisms and in a controlled environment have confirmed that p43 restrains the immune response elicited by IL-13. It signifies that p43 is implicated in overseeing the immune response modulated by IL-13 [94]. Early evidence from experiments using a *Salmonella* reporter system has suggested an effective iron-binding function. Recently, the structure of this distinct sequence, featuring a tail rich in natural histidine, has been identified but not yet published. The p43 might alter the populations of intestinal microflora by impacting the levels of accessible iron, given the site of *Trichuris* infection and its interaction with the gut. Studies were carried out to explore the function of p43 in controlling the growth of intestinal microbes in a laboratory setting, indicating a marked increase in growth rate with the inclusion of p43 in the culture medium, using samples from both unexposed and infected mice [26,105,110].

It is possible that the worm influences microbiome growth to its advantage, potentially through Pp3, or that the microbes respond defensively to the worm and its byproducts. The enhanced growth rate with p43 is probably a result of iron removal from the media, implying that the iron levels in the RPMI assay may not be optimal for some intestinal microbial bacterial strains. Thus, the analysis of iron levels in the growth media, and the adjustment of iron concentration, will be a critical next phase [18,105,110]. Studying the bacterial strains that are expanding as a result of the worms and the presence of p43 may offer explanations for the escalated microbial growth rate. On the other hand, it is equally interesting to speculate that p43 could be involved in activities other than iron sequestration, contributing to the observed enhancement in microbial growth. There is a significant amount of knowledge yet to be gained regarding the multiple functions of p43, and a more in-depth analysis will revolutionize the study of helminths [105,110,111].

**Figure 5:**
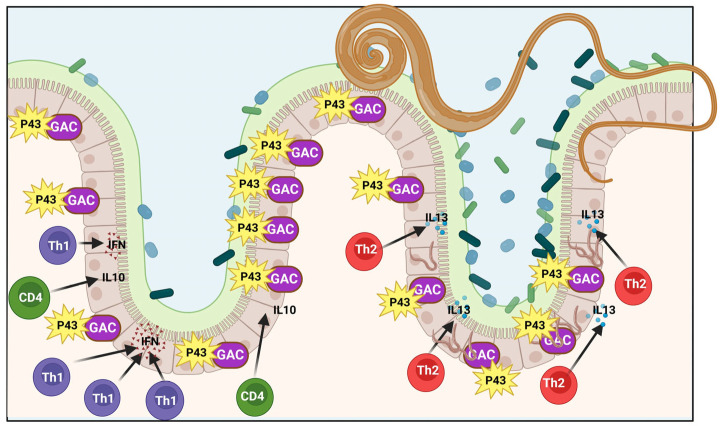
Regulation of the immune response that safeguards against *T. muris* infection. The production of the protein p43 by mature and fully grown worms is intricately associated with its engagement and interaction with glycosaminoglycans, as well as the cytokine known as IL-13, which plays a significant role in this biological process. Shown here is a lone adult worm, with its front end thoroughly embedded in the epithelial surface, while the back end, which is bulbous in shape, is left free and unconfined within the lumen of the structure. The ongoing struggle against chronic infections is largely governed by the activity of interferon-gamma (IFN-γ), which is adeptly controlled by CD^4+^ T helper cells that diligently secrete interleukin-10 to maintain a balanced immune environment. Adult worms produce a large amount of p43, which is located in their specific environment and attaches to the extracellular matrix, for instance. The invasion of L1 larvae by *T. muris* leads to successive infections that trigger a Th2 response and boost IL-13 synthesis. With a high binding affinity, GAG-bound p43 will attach to IL-13 and obstruct its effector functions. Ultimately, this results in a delay and a weakened immune response in the host, possibly increasing the likelihood of parasite survival. This figure was generated using BioRender software version 04.

The complex interplay between the various bacterial species in the gut microbiome affects the stability of the established homoeostatic state. Effective iron chelation compounds are known to be vital for bacteria, affecting their competitive interactions and ability to establish themselves in various infection environments [108,109,110]. Modifications in the ecosystem resulting from *T. muris* infection, including the generation of substances like p43 that influence vital bacterial nutrients like iron Fe^2+^, can establish a stable connection between the parasite and host near the worm. There are certainly other instances of nematodes residing in the digestive tract that can impact the communities of gut bacteria [22,105] (Figure 5).

## 4. Conclusions

The scientific community is highly intrigued by the interaction between roundworms and microbiota, leading to a surge in research activity. In the last ten years, researchers have dedicated significant attention to this subject. It can be hard to determie whether the links are due to the roundworm affecting the microbiota, the microbiota affecting the roundworm, or a mix of both. Therefore, further research is required to elucidate the nature of these connections. Recent studies indicate that analyzing the microbiota may have the potential to forecast the results of certain roundworm infections like ascariasis. Furthermore, new data from experimental investigations could advance our knowledge of the mechanisms behind the interactions between roundworms and microbiota, such as research that reveals the genetic factors of the host that are involved.

The cross-linking mechanisms described here are essential factors influencing the pathogenesis of infections from other notable pathogens. Current evidence confirms that helminth infections can modify the microbiome, while the microbiome can influence helminths directly. These interactions are contingent upon the host’s immune response and have significant implications for host physiology, homeostasis, and the development of disease. Our study concludes that iron is vital for preserving the balance of the internal environment to prevent ailments such as anemia and hereditary hemochromatosis, in addition to influencing the makeup and quantity of gut microbiota. Changes in the gut microbiota composition can significantly affect gut immune responses and the onset of host-related diseases, in addition to modulating the development of the central nervous system. Therefore, P43 functions as an essential asset in the arsenal of helminths, facilitating their ability to regulate mineral accessibility in the mammalian gut and the conditions of the gut microbiome. It is essential to explore how this element affects the early phases of worm infection, particularly in terms of survival and gut attachment.

## Data Availability

Data were gathered from English-language scientific publications using different combinations of the following keywords: ‘Parasites’, ‘Bacterial iron regulatory’, ‘microbial Metabolite’, ‘microbial products’, ‘Gut Microbiome’, ‘bacterial products’, ‘Entomopathogenic nematodes (EPNs)’, ‘Gastrointestinal tract’, ‘*C. elegans*’ as keywords in search queries of different databases and electronic search engines.

## Abbreviations

The following abbreviations are used in this manuscript:

EPNs	Entomopathogenic nematodes
IUS	Iron uptake system
GI	Gastrointestinal tract
LD	Linear dichroism
PO	Pellicula Ovi
PBM	Permeability barrier membrane
EdPC	Electron-dense parietal coating
ESP	Excretory/secretory products
BF	Body fluid
ASABF	*A. suum* antibacterial factor
ROS	Reactive oxygen species
FAC	Ferric ammonium citrate
mtROS	Mitochondrial reactive oxygen species
SOD	Superoxide dismutase
NOX	tissue-specific NADPH oxidase
BIAS	bacterial iron acquisition systems
ESRE	Ethanol and Stress Response Element
IL-13	Interleukin-13
GAC	Glycosaminoglycans
